# High-precision, large-domain three-dimensional manipulation of nano-materials for fabrication nanodevices

**DOI:** 10.1186/1556-276X-6-473

**Published:** 2011-07-27

**Authors:** Rujia Zou, Li Yu, Zhenyu Zhang, Zhigang Chen, Junqing Hu

**Affiliations:** 1 State Key Laboratory for Modification of Chemical Fibers and Polymer Materials, College of Materials Science and Engineering, Donghua University, Shanghai 201620, China

**Keywords:** nano-probe, ZnS nanowire, manipulation, TEM-STM, nanodevices

## Abstract

Nanoscaled materials are attractive building blocks for hierarchical assembly of functional nanodevices, which exhibit diverse performances and simultaneous functions. We innovatively fabricated semiconductor nano-probes of tapered ZnS nanowires through melting and solidifying by electro-thermal process; and then, as-prepared nano-probes can manipulate nanomaterials including semiconductor/metal nanowires and nanoparticles through sufficiently electrostatic force to the desired location without structurally and functionally damage. With some advantages of high precision and large domain, we can move and position and interconnect individual nanowires for contracting nanodevices. Interestingly, by the manipulating technique, the nanodevice made of three vertically interconnecting nanowires, i.e., diode, was realized and showed an excellent electrical property. This technique may be useful to fabricate electronic devices based on the nanowires' moving, positioning, and interconnecting and may overcome fundamental limitations of conventional mechanical fabrication.

## Introduction

The main driving engine of the IT revolution has been geometrical miniaturization of transistors. This has been accomplished with a striking development in microfabrication technology, referred to as "Moore's law", i.e., the number of transistors on an integrated circuit (IC) doubles every 2 years, and industrial guidelines enable multiple devices to be integrated within a given chip area [1,2]. For the past decade, however, the size of the microchips has remained roughly constant and we are approaching the atomic limit of a critical size. Clearly, this evolution cannot continue down this same path much longer. In reality, they are not likely to replace the ordinary transistors, but they may well provide the paradigm shift that will extend "Moore's law". One-dimensional nanoscaled materials, nanowires (NWs), nanotubes (NTs), or even composite nanowires made up of different materials represent attractive building blocks for hierarchical assembly of functional nanoscale devices, which can exhibit a real device with diverse performances and simultaneously function as the "wires", i.e., they can access and interconnect devices that could overcome fundamental limitations of conventional fabrication [3-7]. These unique properties and the intrinsically miniaturized dimensions of NWs' and NTs' building blocks may facilitate the continuation and extension of Moore's law and the evolutionary demand for even faster and smaller electronics in the future. So far, Ferry has developed so-called circuit cleverness by replacing field effect transistor (FET) of vertically oriented structures with parallel nanowires transistor [8]; it can be a great potential to create reconfigurable architectures in which the connections between different functional blocks are changed by switching just a few of the vertical transistors. Indeed, there has been considerable interest in multilayer electronics to offer a more efficient interconnection and processing of digital information. However, existing technologies for creating reconfigurable architectures are limited, such as chemical vapor deposition (CVD) process [9,10], ion etching [11,12], lithography [13-16], fluidic [17], and Langmuir-Blodgett technique [18], which are difficult with high-precision and large-domain three-dimensional (3D) manipulation of the nanowires for nanodevices fabrication with reconfigurable architectures. So, the developing new technique for achieving true 3D-integrated circuits based on the conventional complementary semiconductor transistor technology is critical and remains a challenge.

Herein, we develop a useful technique for high-precision, large-domain 3D manipulation of semiconductor nanowires for fabrication nanodevices, which was performed using a new scanning tunnel microscope (STM) - transmission electron microscope (TEM) holder commercialized by Nanofactory Instruments AB (Gothenburg, Sweden). We selected Sn-tipped, tapered ZnS semiconductor nanowires as a manipulative material, which were prepared via a Sn-catalyzed vapor-liquid-solid (VLS) growth process, as described in our previous reports. In our study, we innovatively fabricated semiconductor nano-probes of tapered ZnS nanowires through a melting and solidifying by electro-thermal process; and then the ZnS nanowires are manipulated by as-prepared nano-probes after a sufficiently electrostatic force is initiated between the probes and nanowires by direct current (DC) bias voltage, in which we can finely move individual nanowires at the desired location, with precisely controllable position and moving direction in 3D scale. With some advantages of high precision (with an accuracy of approximately 0.5 nm) and large domain (reaching 1 cm), the present manipulation technology could be used to realize top-down fabrication of nanostructure nanodevices made of three nanowires, which demonstrates good electricity properties and could be used in FET with vertically structure and other electronic devices. This present nanoscaled manipulation technology shows a new opportunity for fabrication nanodevices with a special structure, excellent properties, and potential applications.

## Experimental

### Sample Preparation

ZnS, SnO, SnO2, and activated carbon powders were purchased from Sinopharm Chemical Reagent Co., Ltd. The tapered ZnS nanowires tipped with a spherical Sn particle were synthesized via a Sn catalyzed VLS growth process in a horizontal high-temperature resistance furnace. Briefly, a graphite crucible containing a mixture of ZnS (1.5 g), SnO (0.3 g), SnO_2 _(0.2 g), and activated carbon powders (0.1 g) was placed in the central zone of a quartz tube, which was heated to 1,150°C at a rate of 10°C min^-1^, kept at this temperature for 4 h, and then cooled to room temperature. The whole process was carried out under a constant flow of pure N_2 _at a rate of 450 mL min^-1^. Finally, the products were collected from the inner wall of the tube for our following STM-TEM manipulations.

### Three-dimensional manipulation Study

The manipulations of as-prepared ZnS nanowires were carried out using a new STM-TEM holder commercialized by Nanofactory Instruments AB, which was arranged within a 200 kV field emission high-resolution TEM (HRTEM; JEM-2010F, JEOL Ltd., Tokyo, Japan). A gold (Au) cantilever was attached to a fixed electrical sensor, whereas a platinum (Pt) cantilever was placed on the piezo-movable side of the holder. At the beginning, relative positions between Pt and Au cantilevers were manually adjusted with tweezers under an optical microscope in order to obtain a minimal possible gap between them, which can be distinguished by eyes. Then, the *X*, *Y*, and *Z *positions of two cantilevers were adjusted through the nanoscale precision piezo-driven manipulator inside the TEM, so as to make horizontal between two cantilevers. A PC-compatible software automatically coordinates the final stages and controls the nanowire displacement and movement rate. All experiment processes are video recorded in real-time using a TV-rate charge-coupled device camera with a 30 frames per second recording speed. On the basis of the model adopted from the classical electricity, the electrical properties were evaluated by the dedicated software and electronics from Nanofactory Instruments AB.

## Results and discussion

**Figure 1:**
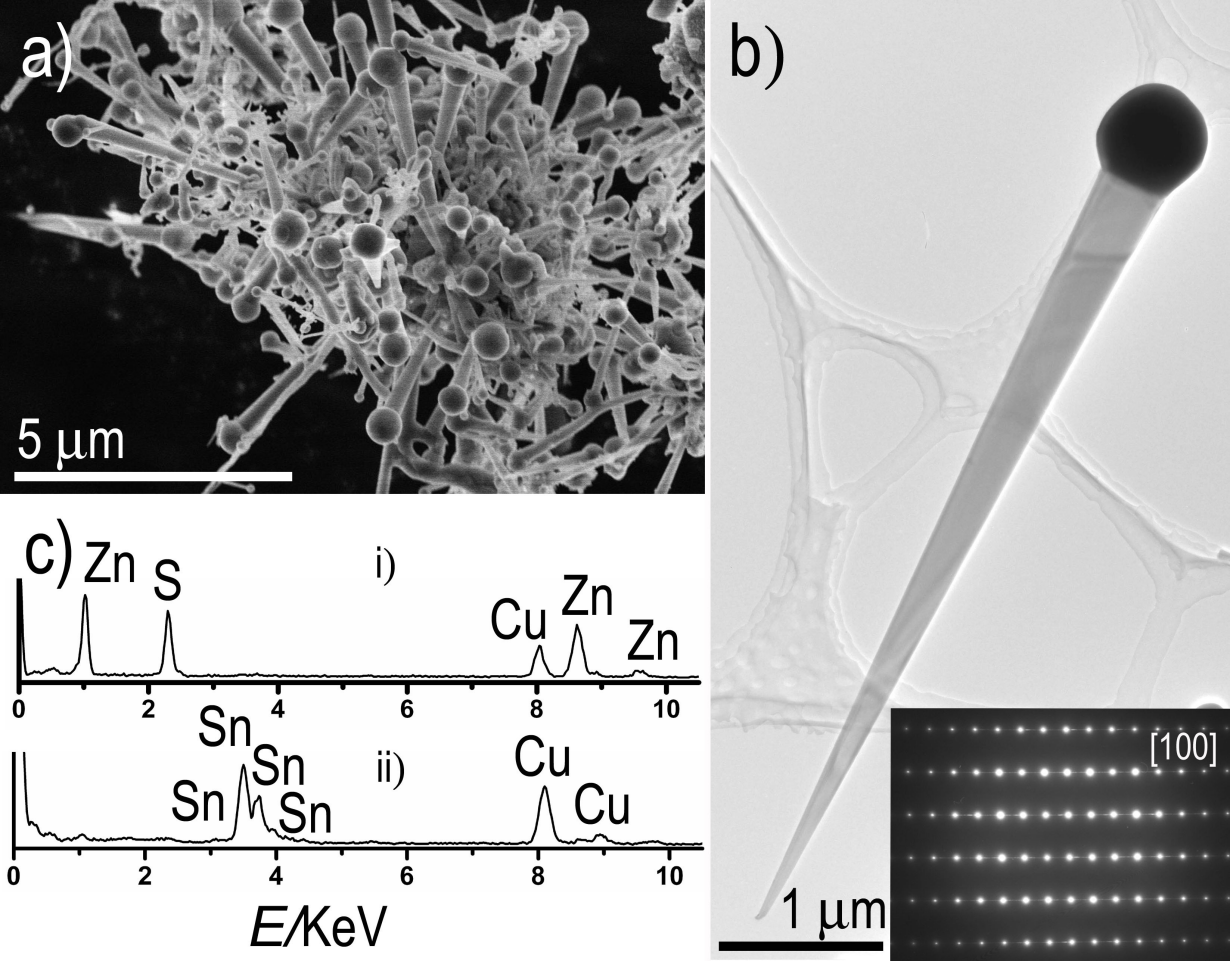
**SEM image, TEM image and the corresponding ED pattern, and EDS spectra of nanowires**. (**a**) SEM image of as-grown tapered ZnS nanowires. (**b**) TEM image showing a tapered ZnS nanowire tipped by a Sn particle on its thicker end. The lower-left inset showing the corresponding ED pattern recorded with an incident electron beam along the [100] direction. (**c**) EDS spectra recorded for a nanowire (curve i) and a spherical Sn particle on its end (curve ii).

The tapered ZnS nanowires were synthesized at 1,150°C via a Sn-catalyst VLS growth using a mixture of ZnS and SnO powders as starting materials. As shown in a scanning electron microscopy (SEM; S-4800, Hitachi Co., Tokyo, Japan) image (Figure 1a) many tapered nanowires with a spherical particle at their tip ends were formed in the product. Typically, they are straight and have a length ranging from several to tens of micrometers. TEM image reveals that as-grown nanostructures are in fact ZnS nanowires with Sn spherical particles at their tip ends. The corresponding electron diffraction (ED) pattern (the lower-right inset in Figure 1b) can be indexed as the [100] zone axis diffraction pattern of the wurtzite ZnS single crystal. Normally, the diameters of these ZnS nanowires gradually decrease and become smaller and smaller along their lengths, from 200-400 nm at the thicker end to 30-80 nm at the thinner end, respectively (Figure 1b); the spherical Sn particles on the thicker end have several hundred nanometers in diameter. The energy-dispersive spectra (EDS) shown in Figure 1c were recorded for nanowire part (curve i) and Sn particle part (curve ii), respectively, confirming that the nanowire has a chemical composition of ZnS, and the particle is metallic Sn.

**Figure 2:**
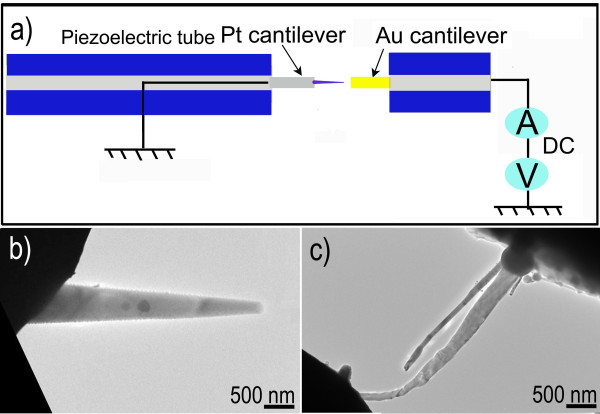
**Experimental setup and ZnS nanowire nano-probe**. (**a**) Schematics of the experimental setup within a STM-TEM holder. (**b**) The tapered ZnS nanowire nano-probe. (**c**) The ZnS nanowire nano-probe bridged between the Au and Pt cantilevers.

The nano-probe preparation is performed using the STM-TEM holder within a 200-kV HRTEM. An experimental setup is sketched in Figure 2a. An Au cantilever is attached to a fixed electrical sensor, whereas a Pt cantilever with an amount of as-grown tapered ZnS nanowires is placed on the piezo-movable side of the holder. At the beginning, the relative positions of two cantilevers are manually adjusted with tweezers under an optical microscope to get a minimal possible gap between them. Then, the *X*, *Y*, and *Z *positions of the Au cantilever and individual tapered ZnS nanowires are adjusted through the nanoscale precision piezo-driven manipulator of STM-TEM holder, to make a tapered ZnS nanowire bridge between two cantilevers. On the basis of the classical electricity, the electrical properties of this bridged ZnS nanowire have been evaluated by the dedicated software and electronics from Nanofactory Instruments AB. An individual tapered ZnS nanowire (tipped with a spherical Sn particle) is built to serve as nano-probe. The tipped Sn particle on the thicker end of ZnS nanowire contacts with the Pt cantilever, whereas the thinner end of this ZnS nanowire contacts the Au cantilever, followed by applying a bias voltage of approximately 10 V. Once a current passes through this Sn-tipped ZnS nanowire, because of a resistance of the contact point between the Sn particle and the Pt cantilever, a local temperature increase due to Joule heating can be generated. It is known that the melting point of nanostructured materials can be much lower than that of their bulky counterparts (for example, the difference between the melting point of Au nanoparticles and Au bulk material is over 400°C) [19-21]. So, it is reasonable to assume that the melting point of the Sn nanoparticle is also lower than that of a Sn bulky material (bulk Sn: m.p. of 232°C) [22]. Consequently, the Sn particle may entirely melt (even though the melting point of bulk Sn is far above the room temperature) at a very low basic pressure (approximately 1 × 10^-5 ^Pa) in the TEM chamber. This leads to a successful welding of the spherical Sn particle onto the Pt cantilever and the thicker end of the ZnS nanowire part, while the thinner end of the ZnS nanowire and the Au cantilever are keeping a perfect physical contact. As a result, the ZnS nanowire nano-probe has been realized when the Sn welding is solidified with the bias voltage unloaded and the local temperature decreased, as shown in Figure 2b. Shown in Figure 2c is the ZnS nanowire nano-probe bridged between the Au and Pt cantilevers.

Here, let us consider the principle of the electro-thermal system of this ZnS nano-probe where the thicker and thinner ends of ZnS nanowire contact with the Sn particle and Au cantilever, respectively. When applying a bias voltage, a constant direct current *I *flows through this nano-probe system. The rate of heat quantity generated (per unit time) on the probe with a length *ΔX *is given by *Q = I^2^ΔR*. The term *ΔR*, the electrical resistance of the segment *Δx*, is given by *ρΔx/ΔA*, where *ρ *is the electrical resistivity of a given material, and *ΔA *is a cross-sectional area of the segment. Figure 2c schematically shows the tapered ZnS nanowire nano-probe inside the STM-TEM. The measured resistance of this nano-probe system, *R*_m_, can be expressed as: *R*_m _= *R*_Pt _+ *R*_Au _+ *R*_NW_, where the *R*_Pt _is the contact resistance between the Pt cantilever and Sn particle, *R*_Au _is the contact resistance between the Au cantilever and ZnS nanowire tip, and *R*_NW _is the intrinsic resistance of ZnS nanowire. In fact, the value of *R*_Au _becomes larger compared with that of *R*_NW_, because the area of the actual contact surface is considerably small; similarly, the value of *R*_Pt _is large compared with that of the Sn particle. Therefore, the corresponding values of *Q*_Au _and *Q*_Pt _around their contact regions are both larger than those on ZnS nanowire and Sn particle, giving rise to a local temperature increase. But, Sn particle is metallic conductor, with a lower resistance than that of ZnS nanowire, and *Q*_Pt _is lower than *Q*_Au_. For metals, *Q *is temperature dependent, and is usually given by *Q *= *Q*_0_[1 + *a*(*T *- *T*_0_)], where *Q_0 _*is the electrical resistivity at a reference temperature *T*_0_, and *α *is a temperature coefficient of the resistance and *T *is the temperature, which is normally positive for metals. The increase of local temperature at the contact region of the Sn particle leads to an increase of *ΔR*, and this further accelerates the temperature increase. Due to the high contact, electrical resistance (of the ZnS nanowire and Sn nanoparticle) with a low melting point (as discussed earlier) will make the Sn nanoparticle to be locally melted by a current. This will cause the Sn particle weld onto Pt cantilever and form a perfect physical contact between Pt cantilever and Sn particle. On the contrary, because ZnS nanowire has a high melting point (about 1,650-1,900°C) [23], welding process cannot occur at the contact region of the ZnS nanowire and Au cantilever, even if a local temperature increases at this region. After realization of this perfect physical contact, the electrical resistance at the contact region between Pt cantilever and Sn particle decreases, and then the amount of heat generated at the region decreases, and the local temperature at this region will drop. The local temperature at this region is so low that the contact region will be solidified, and the ZnS nanowire nano-probe is realized.

**Figure 3:**
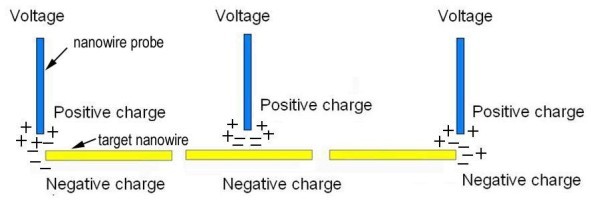
**Schematic of the setup shows moving nanomaterials by electrostatic force**. A DC bias voltage is applied between the nanowire probe and the target nanomaterials, and an electrostatic force between the nanowire probe and the target nanomaterials is initiated.

**Figure 4:**
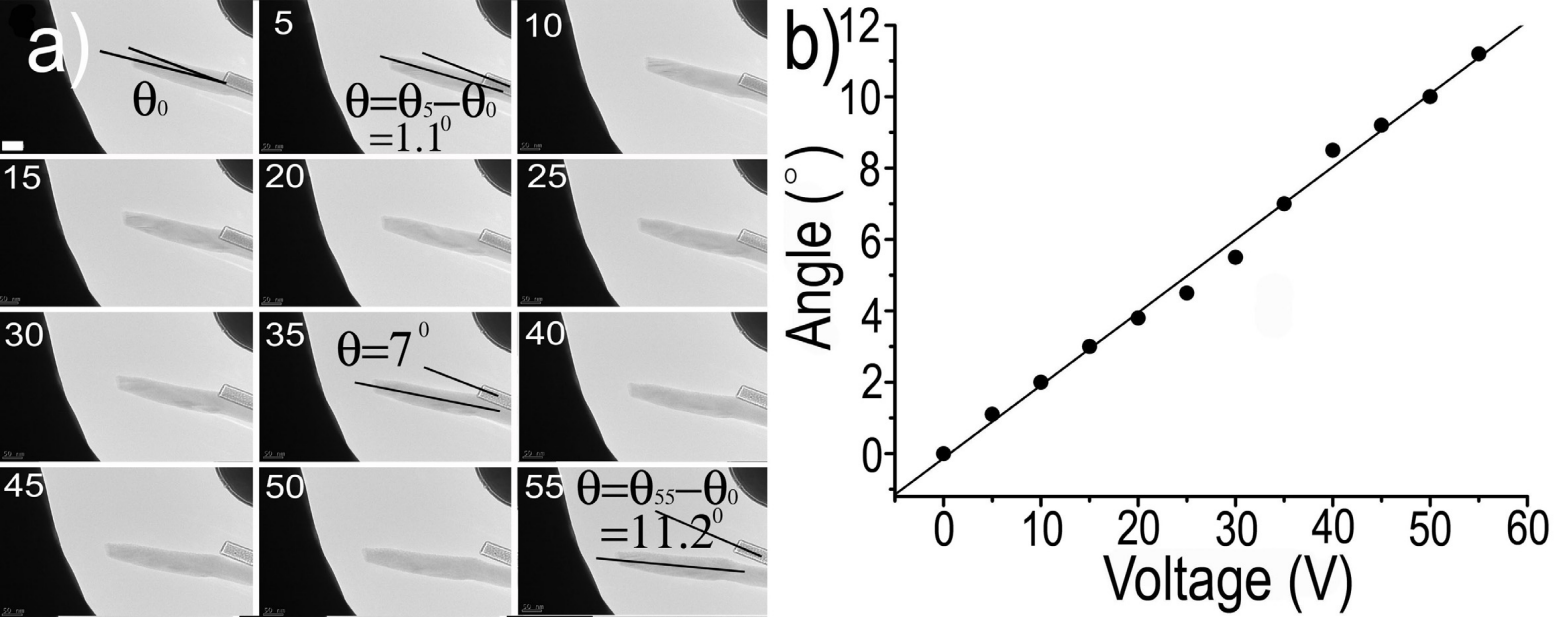
**TEM images and plot of the angle between ZnS nanowire probe and a marked nanowire**. (**a**) Consecutive TEM images shows continuously the bending of the ZnS nanowire probe, continuously increasing DC bias voltage from *V *= 0 V to *V *= 55 V. (**b**) A plot of the angle between the ZnS nanowire probe and the marked nanowire and applied voltage over time.

Employing the ZnS nanowire nano-probe, 3D scale high-precision manipulating semiconductor nanowires for the fabrication of prototypical nanodevices is achieved, and a possible mechanism for this manipulating process is proposed in Figure 3. When a sufficient DC bias voltage is loaded between the ZnS nanowire probe and an adjacent target nanowire (to be moved) on opposite cantilever, electrostatic force is initiated. If the nanowire probe has some positive electric charges, the target nanowire on opposite cantilever will have negative charges. Oppositely, if the nanowire probe has negative electric charges, the target nanowire on opposite cantilever has positive electric charges. Also, the ZnS nanowire probe can be located at different positions on the target nanowire, and then electrostatic force can be initiated at different positions on the target nanowire. The strong adhesion of the target nanowire to the ZnS nanowire probe will be effectively formed when the ZnS nanowire probe contacts it by electrostatic force. So, we can accomplish a simple, high-precision, and large-domain movement of some nanowires for fabricating nanodevices. These TEM images show the ZnS nanowire probe attached to the Pt cantilever and an Au electrode (or cantilever) oriented opposite. An obvious feature showing achieving reliable electrostatic force can be demonstrated from the consecutive variation of an angle between the ZnS nanowire probe and a marked nanowire (marked by two lines). As the bias voltage increases from 0 V, electrostatic force occurs and the ZnS nanowire probe bends toward the opposite Au electrode. Continuously increasing bias voltage results in a continuous increase of the ZnS nanowire probe bending degree. A series of TEM images of the ZnS nanowire probe continuously bending when the bias voltage is continuously increased from *V *= 0 V to *V *= 55 V are shown in Figure 4a. As the DC bias voltage increases, the electrostatic force increases, and the ZnS nanowire probe bending is clearly observable. The angle between the nanoprobe and marked nanowire (indicated by two lines) when voltage applied is 0, 5, 35, and 55 V, is 0°, 1.1°, 7°, and 11.2°, respectively. A curve of the ZnS nanowire probe bending angle and applied voltage over the whole process is shown in Figure 4b, which has also been acquired using counter bias voltage. It illustrates that the angle of the nanowire probe bending is associated with the applied DC bias voltage. In addition, the electrostatic force of other materials, such as, Si nanowires, was confirmed in our study (see Figure S1 in Additional file 1). The present finding suggests that an electrostatic force motivated by the applied bias voltage is a common phenomenon for moving nanomaterials.

**Figure 5:**
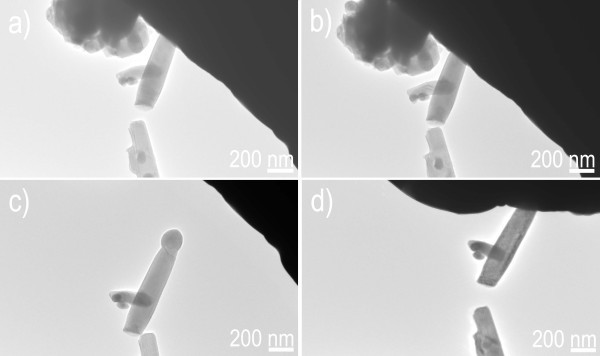
**Manipulating a ZnS nanowire in a form of "left/right geometry"**. (**a**) Moving ZnS nanowire. (**b**) Electrostatic force initiated by a bias voltage. (**c**) Manipulating ZnS nanowire. (**d**) Placing the ZnS nanowire at a desired location.

**Figure 6:**
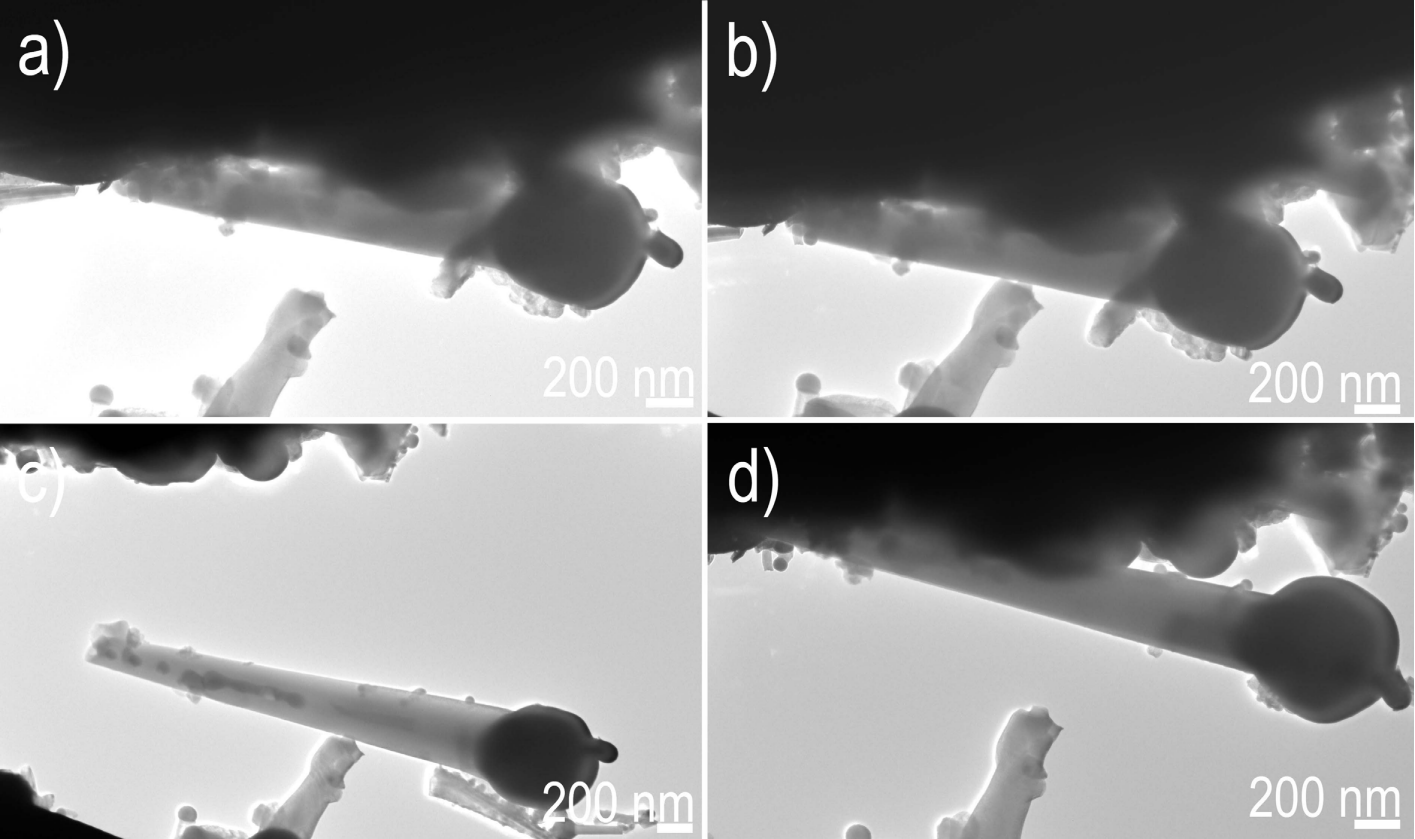
**Manipulating a tapered ZnS nanowire in a form of "middle geometry"**. (**a**) Finding a target tapered ZnS nanowire. (**b**) Electrostatic force launched by a bias voltage. (**c**) Moving the tapered ZnS nanowire. (**d**) Placing the tapered ZnS nanowire at a desired location.

Nano-manipulator for fabrication nanodevices is particularly suitable for industrial applications of localized bottom-up integration due to its nanoscaled spatial resolution, long manipulating distance, no special requirement during the sample process, high efficiency of manipulation and observation, and proven assimilation into production lines. A large quantity of ZnS nanowires, which is dispersed onto Au cantilever, is manipulated by as-fabricated ZnS nanowire probe using the STM-TEM holder. Manipulating left/right geometry of the nanowires, firstly, the target ZnS nanowires were located on Au cantilever by TEM-STM manipulator (Figure 5a). A gentle force pushes the nanowire probe to approach the tip of the target ZnS nanowire (left/right geometry of the nanowires) by TEM-STM manipulator. Then a 9-V DC bias voltage is applied between the ZnS nanowire probe and the target ZnS nanowire on Au cantilever, initiating the electrostatic force. The close connection between the ZnS nanowire probe and target ZnS nanowire occurs using the initiated electrostatic force when the ZnS nanowire probe touches the target ZnS nanowire, as shown in Figure 5b. The target ZnS nanowire is then lifted from the Au cantilever, followed by controllable and precise manipulation with an accuracy of 0.5 nm to destination, i.e., TEM-SEM manipulator (Figure 5c). Finally, the DC bias voltage is unloaded by the dedicated software when the target nanowire is released at desired location, as shown in Figure 5d (also see Movie 1 in Additional file 2). By the same way, manipulating the nanowires in middle geometry is also accomplished, as shown in Figure 6. Figure 6a shows the target ZnS nanowire on Au cantilever by TEM imaging. A considerable force was loaded by the ZnS nanowire probe to the middle of the target nanowire. Then an electrostatic force is launched between the nanowire probe and the target nanowire on Au cantilever by applying 15-V bias voltage. The closely adhesion originated from the electrostatic force occurs, and thus the nanowire probe touches the target ZnS nanowire. The target ZnS nanowire is then lifted from the Au cantilever by electrostatic force (Figure 6b), followed by precise moving controlled by TEM-STM manipulator (Figure 6c). Finally, the bias voltage is unloaded by the dedicated software when the target ZnS nanowire is precisely placed at the desired location (Figure 6d) (also see Movie 2 in the Additional file 2). By fabricating the nanowire probe and applying electrostatic force, the present manipulation technology for creating reconfigurable architectures displays a high precision with an accuracy of 0.5 nm and a 3D-scaled large domain reaching 1 cm, to make the nanodevices with complex structures possible; also, the whole manipulating process is clean, simple, quick, and reliable; it avoids the disadvantages of a long time, high expense, and contaminations involving other methods such as plasma etching, lithography, and so on.

By simply applying a specific voltage and producing an electrostatic force, the nanowires of left/right geometry have been manipulated, but it seems a bit difficult to manipulate nanowires in a form of the middle geometry. In order to exclude any exceptional events, a systematic investigation of the movements of many nanowires is carried out by the same method. Table 1 presents the results from 45 various positions of the ZnS nanowires. In the case of "left/right geometry" of the nanowires, all 17 attempts are successful without an exception, but one attempt is fail to be manipulated when the nanowires with middle geometry are randomly chosen. Without applying voltage, all 17 attempts to manipulate nanowires in the "left/right/middle geometry" of the nanowires are failed. The results in Table 1 therefore, give an important indication that the electrostatic force from DC bias voltage is the key factor for manipulating individual nanowires. In addition, two reasonable reasons that these nanowires in a form of middle geometry are failed to be manipulated in our attempts can be illuminated as follows. On the one hand, an electron beam is focused on the compressed ZnS nanowires on the Au cantilever during the sample preparation process, and the strong adhesion of the target ZnS nanowires to the Au cantilever has been greatly increased. The required threshold value by the DC voltage gets larger than that of other nanowires on the Au cantilever, which corresponds to the activation energy for manipulating nanowires. On the other hand, Au cantilever could be somewhat molten by Joule heating when applying a specific voltage for a long time. The target ZnS nanowires are possibly embedded in Au cantilever and closely connecting among them should be enhanced. So, in order to availably manipulate nanowires, we should increase electrostatic force by increasing DC voltage to manipulate nanowires instead of a relatively stationary voltage.

**Table 1:** Statistics for the various positions of the ZnS nanowires

Voltage	Geometry	Number of attempts	Number of successful	Description of failure
Yes	Left/right	17	17	0
	Middle	11	10	1
No	Left/right	9	0	9
	Middle	8	0	8

Most importantly, semiconductor ZnS nanowire probe replacing common metal probe has been also successfully applied for manipulating low-melting point metals and ultrathin metal nanowires. Referring to welding of ultrathin Au nanowires, atomic diffusion and surface relaxation are obvious in the aforementioned nanoscale process [24-26]. It is well recognized that the diffusion barrier for a single metal atom on the ultrathin metal surface is quite low (typically less than 1 eV) [27]. Thermal activation, even at room temperature, is enough to overcome such low barriers, so isolated metal atoms can diffuse rapidly by means of surface diffusion. The mechanical manipulation can clearly provide the necessarily extra driving force to facilitate the cold welding and unification of two nanowires. Additionally, because of low-melting point metals and ultrathin metal nanowires, current flowing through nanostructured metals faces a major problem that the nano-objects can be structurally and functionally damaged due to atom migration due to Joule heating and electromigration [28,29]. Here, we innovatively have developed the nano-probes of semiconductor nanowires and excellently solved these mentioned problems.

**Figure 7:**
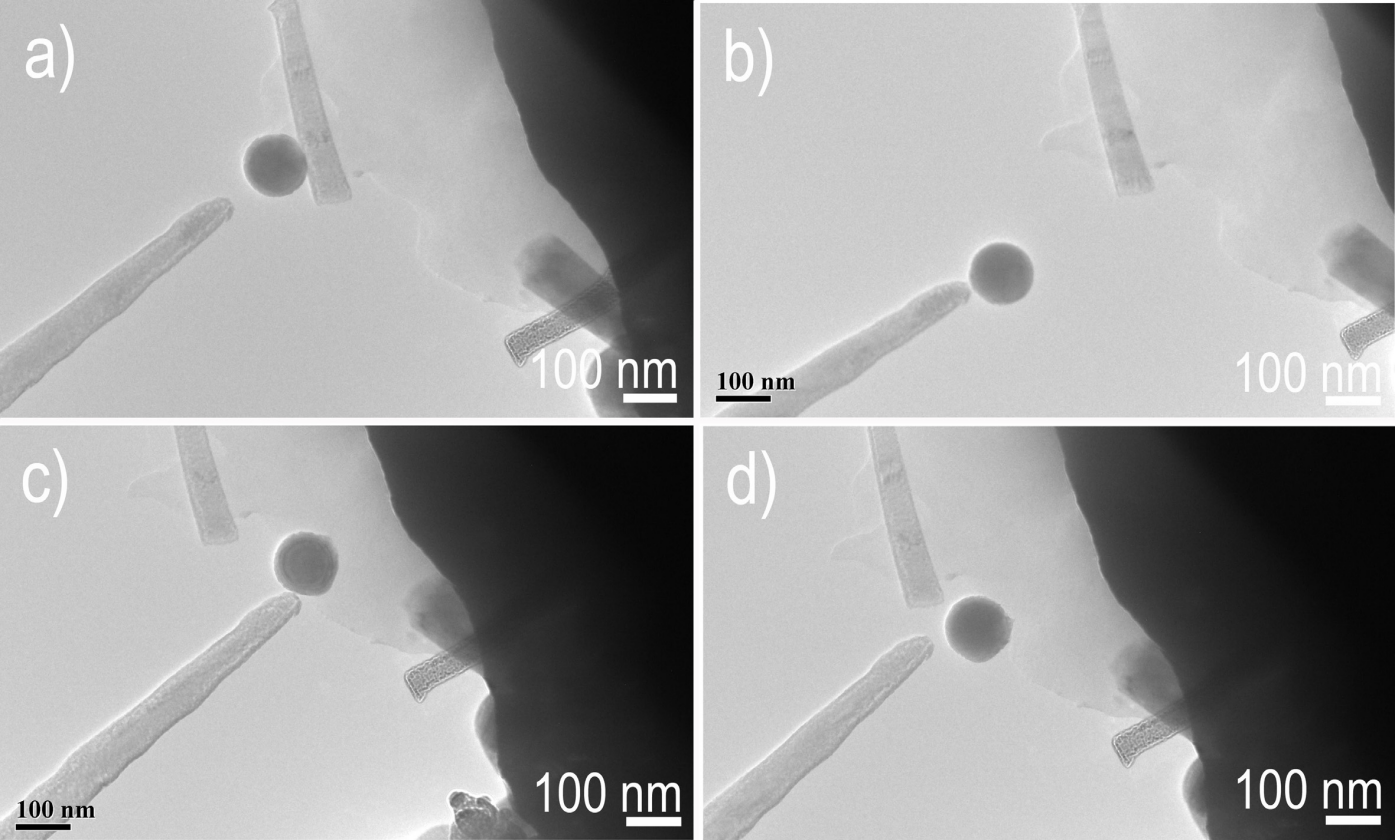
**Manipulating low-melting point metal Sn nanoparticle by the ZnS nanowire probe**. (**a**) Approaching Sn particle. (**b**) Electrostatic force launched by a DC bias voltage. (**c**) Moving Sn particle. (**d**) Placing Sn particle at a desired position.

**Figure 8:**
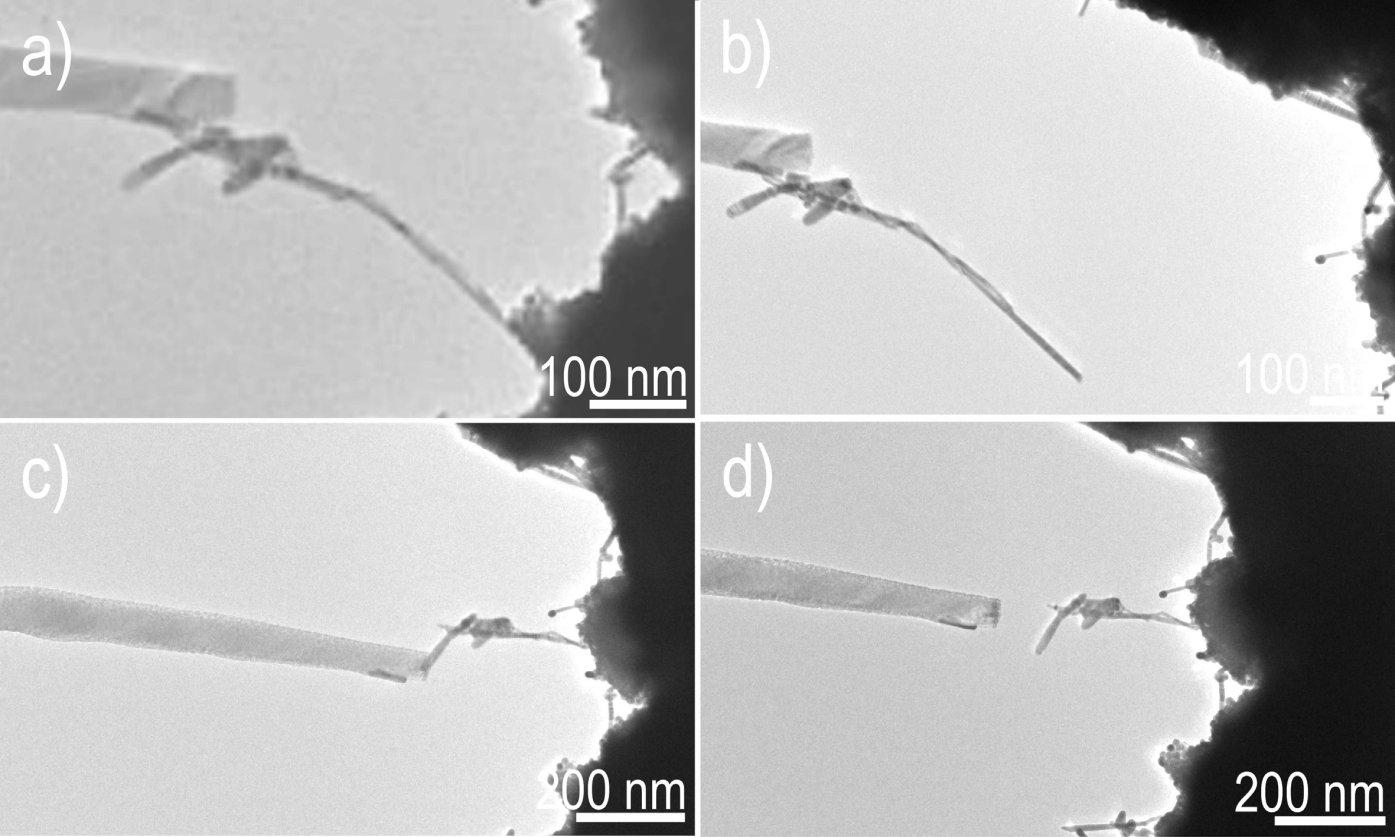
**Manipulating ultrathin Au nanowire by the ZnS nanowire probe**. (**a**) Approaching ultrathin Au nanowire. (**b**) Electrostatic force launched by a DC bias voltage. (**c**) Moving ultrathin Au nanowire. (**d**) placing ultrathin Au nanowire at a desired position.

Sequential TEM images illustrate the process of manipulating low-melting point metal Sn nanoparticle (approximately 100 nm in diameter) with the nanowire probe of the ZnS semiconductor (Figure 7) [30]. The as-prepared nanowire probe has a diameter of approximately 32 nm near the cap. These Sn nanoparticles (with 50 nm in diameter) are dispersed onto Au cantilever (Figure 7a). As shown in Figure 7b, the as-fabricated nanowire probe approaches to Sn particle, then the electrostatic force is launched between the nanowire probe and a Sn particle on Au cantilever by applying 8 V DC bias voltage. The Sn particle is adsorbed on the probe when the nanowire probe connects the Sn particle. The adhering of the junction between the target Sn particle and probe has been further improved by moving the probe back and forth, (by changing ZnS nanowire probe through TEM-STM), and then manipulated from the Au cantilever (Figure 7c). Finally, after the Sn particle is precisely put at the desired location, the DC bias voltage is unloaded by the dedicated software (Figure 7d). We would like to clarify key points that can be deduced from the above experiments shown in Movie 3 (in Additional file 2). The Sn particle is structurally and functionally undamaged, or no welding occurred between the Sn particle and ZnS nanowire probe, even under the conditions of atom migration from Joule heating and electromigration in high current. By the same method, ultrathin Au nanowires have also been successfully manipulated, as shown in Figure 8. Figure 8a shows the movement of Au nanowires (also see experimental section in Additional file 1) (with approximately 10 nm diameter) on Au cantilever [31]. A gentle mechanical force pushed by the nanowire probe was exerted to the tip of Au nanowire, controlled by TEM-STM manipulator. A 4-V DC bias voltage is applied between the nanowire probe and ultrathin Au nanowire, and then, an electrostatic force is generated. The selected Au nanowire is then lifted from the substrate by the electrostatic force (Figure 8b), and is carefully moved by the TEM-STM manipulator (Figure 8c). The DC bias voltage is unloaded by the software after the nanowire is placed at the designated position (Figure 8d) (also see Movie 4 in Additional file 2). Interestingly, although the diffusion barrier of a single metal atom on ultrathin metal surface is quite low, no welding happens between ultrathin Au nanowire and ZnS nanowire probe by atom migration due to Joule heating. So, it is concluded that both low-melting point metals and ultrathin metal nanowires are easily manipulated by our fabricated semiconductor nanowire probe.

**Figure 9:**
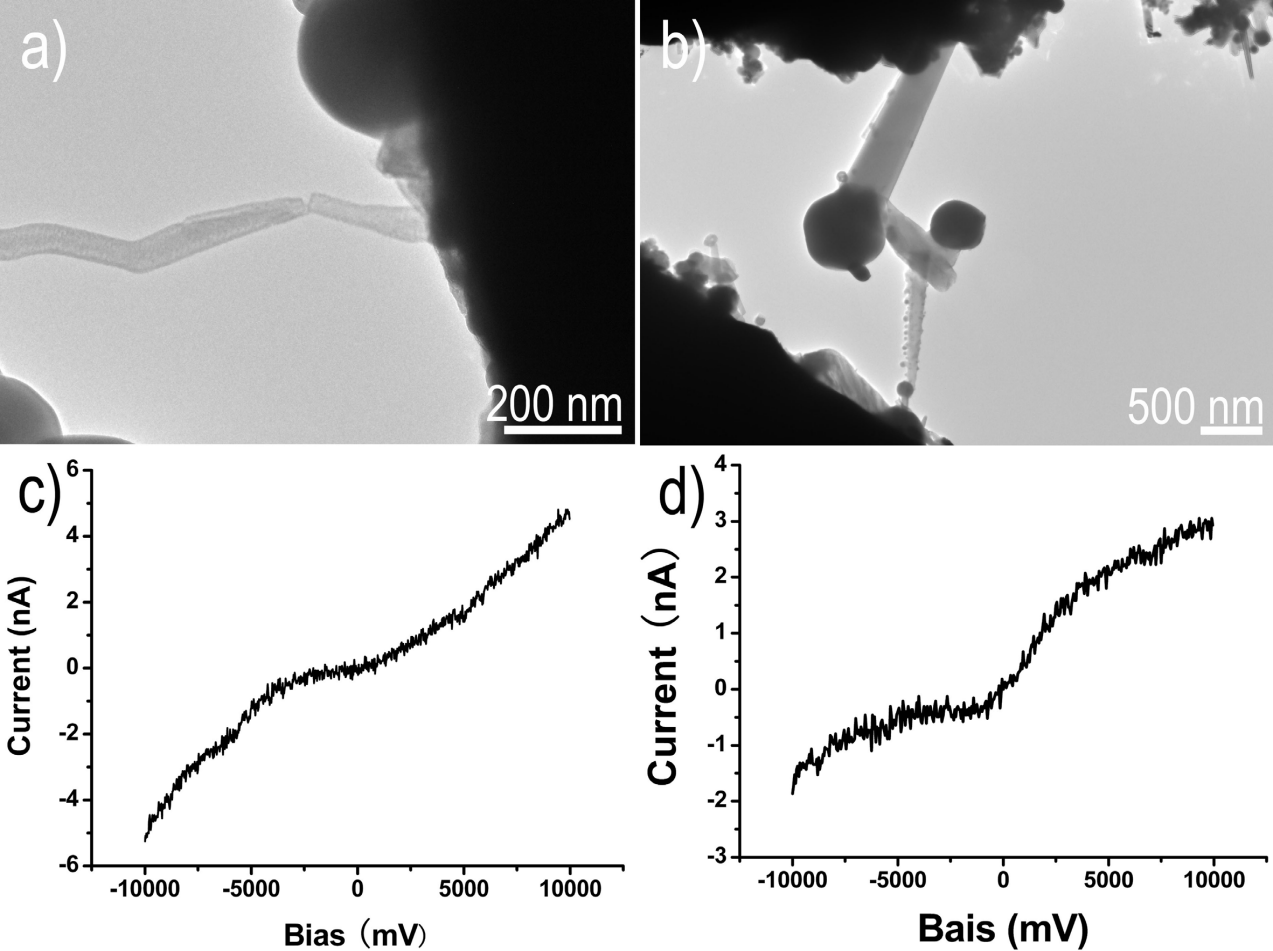
**Jointing three ZnS nanowires and current-voltage characteristic curves**. (**a**) Jointing three ZnS nanowires for fabrication a nanodevice. (**b**) Current-voltage (*I*/*V*) characteristic curves of the three ZnS nanowire device recorded by the STM-TEM holder.

To study the electrical behavior, a diode nanodevice has been constructed by ZnS nanowires, realized by the nanowire probe manipulation. Here, we designed a device consisting of three vertically oriented ZnS nanowires (Figure 9a) which were linked together and "Z" shaped through a welding process by high energy electron beam irradiation [32,33]. The *I-V *curves of the fabricated device are plotted according to the *I-V *data obtained when the voltage is ramped up, as showed in Figure 9b, which are recorded with a voltage ranging from -10 to 10 V for 5,000 ms. ZnS (ZnS: bandgap energy of E_a _is approximately 3.66 eV) nanowires with a lower work function than those of Pt (Φ is approximately 5.1 eV) and Au (Φ is approximately 5.65 eV) makes a Schottky contact between Pt and Au. As shown in Figure 9b, eight cycles were performed for this device made of three ZnS nanowires and the *I-V *curves show typically Schottky characteristic. An excellent stability of ZnS nanowires nanodevice was indicated from the eight circles, and a current value was varied from -2.5 to 3 nA with a voltage ranging from -10 to 10 V, which clearly demonstrates that the electrical resistivity changed very little within each circle. The three contacts to the ZnS nanowires in every heterojunction are found to behave as two diodes connected in series face-to-face, which exhibits the *I-V *characteristics of a Schottky diode-like characteristic. By comparison, under the same condition as that of the device consisting of three ZnS nanowires (Figure 9), the current of individual ZnS nanowires was varied from -12 to 12 nA, as shown in Figure S2 (in Additional file 1). Thus, the current variation range of individual ZnS nanowires is about four times as large as that of three ZnS nanowires device under the same voltage condition. Two factors may be responsible for an increase of the resistance of three ZnS nanowires' device, i.e., (1) intrinsic resistance of the fabricated device of three ZnS nanowires is larger than that of individual ZnS nanowires; (2) the contact regions between ZnS nanowires has higher electrical resistance, and the resistance of two contact regions brings a small current value. The perfect electrical properties of diode nanodevice demonstrate that it is possible to design nanodevices and complex structures by nanowire-probe manipulation technology.

## Conclusions

In summary, we have fabricated a versatile nanoscaled probe with as-synthesized tapered ZnS nanowires with the STM-TEM holder through a melting and solidifying by an electro-thermal process. This fabricated ZnS nanowire probe can precisely, controllably, and 3D-scaled manipulate free-standing nanomaterials including semiconductor nanowires, metal nanowires, and nanoparticles, without structurally and functionally damage, to the desired locations for further making nanodevices. Interesting, by the developed manipulating technique, the nanodevices, i.e., vertical transistors, which were made of three vertically interconnecting nanowires were realized and showed an excellent electrical property, suggesting that it may be useful by the present technique to design and fabricate electronic devices based on nanowires' moving, positioning, and interconnecting. Importantly, this developed nanowire manipulation technology could overcome fundamental limitations of conventional mechanical fabrication and would bring a new opportunity for extending Moore's Law.

## Competing interests

The authors declare that they have no competing interests.

## Authors' contributions

RZ carried out the in-situmanipulation studies and materials synthesis, participated in the sequence alignment and drafted the manuscript. LY, ZZ, ZC participated in discussion of the study. RZ and JH participated in the design of the study and performed the statistical analysis. RZ and JH conceived of the study, and participated in its design and coordination. All authors read and approved the final manuscript.

## Supplementary Material

Additional file 1 **Supporting information: high-precision, large-domain three-dimensional manipulation of nano-materials for fabrication nanodevices**. Supporting Information.doc, 166K. Click here for file

Additional file 2 **Supporting information: movies**. Movie.zip, 2573K. Click here for file
