# Supplementary material for: High-precision, large-domain three-dimensional manipulation of nano-materials for fabrication nanodevices
Source: Nanoscale Res Lett. 2011 Jul 27;6(1):473. doi: 10.1186/1556-276X-6-473 (PMC3211986; doi:10.1186/1556-276X-6-473)
Supplement: Additional file 1 — Supporting information: high-precision, large-domain three-dimensional manipulation of nano-materials for fabrication nanodevices. Supporting Information.doc, 166K. [file 1556-276X-6-473-S1.DOC]

**Supporting Information**

**High Precision, Large Domain Three-dimensional Manipulation of nano-materials for Fabrication Nanodevices**

Rujia Zou, Li Yu, Zhenyu Zhang, Zhigang Chen, and Junqing Hu*


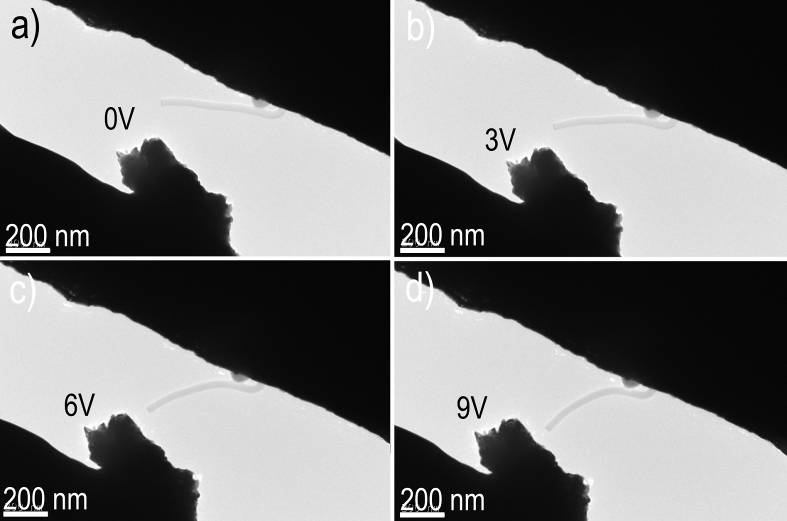


**Figure S1**. Consecutive TEM images shows continuously bending of the Si nanowire, continuously increasing DC bias voltage from *V* = 0 V to *V* = 9 V.


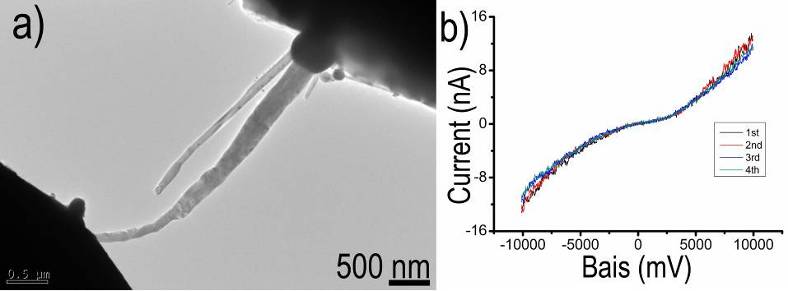


**Figure S2** **a** TEM image shows an individual ZnS nanowire mounted between a platinum and a gold cantilever. **b** Current-voltage (*I/V*) curves are recorded from with the STM-TEM holder. The *I–V* curves of the ZnS nanowire are plotted according to the *I–V* data obtained when the voltage is ramped up, which are recorded with a voltage ranging from -10 to 10 V for 5000 milliseconds. ZnS nanowire makes a Schottky contact between Pt and Au. The four cycles are performed for an individual ZnS nanowire and the *I–V* curves show typically Schottky characteristic.

**Supporting** **experimental section**

The ultrathin Au NWs were synthesized by the reduction of HAuCl_4_ in oleic acid (OA) and oleylamine (OAm). Here, OAm serves both as a reducing agent and a stabilizer. In a typical synthesis of micrometer long Au NWs with 5-10 nm diameter (Figure S3), a solution (4 mL hexane and 4 mL OAm) of 0.2 g of HAuCl_4_ was added to the mixture of OA (10 mL) and OAm (8 mL) at 80 °C, with vigorous magnetic stirring under nitrogen atmosphere. Magnetic stirring was stopped after 10 min, and the solution was kept steady at this temperature for 4 h. The dark product was washed several times with ethanol and to remove any hexane residual, and finally dried in vacuum at 60 °C for 12 h for further applied.


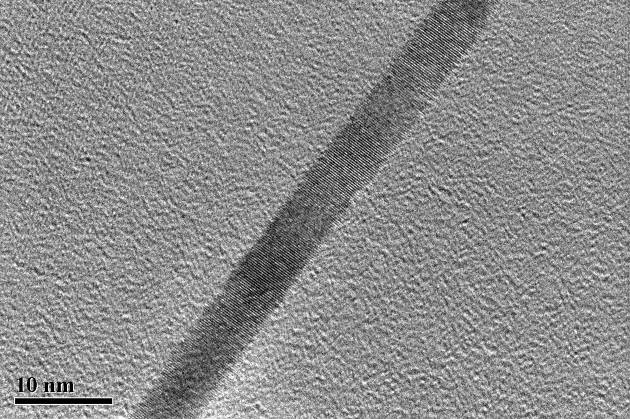


**Figure S3** TEM image of a single ultrathin Au nanowire.
